# The Trigeminocardiac Reflex? Severe Bradycardia Secondary to Facial Trauma: A Case Report

**DOI:** 10.5811/cpcem.43035

**Published:** 2025-07-08

**Authors:** Boris Penev, Hallmon Hughes, Katherine Scarpino, Daniel J. Ritter

**Affiliations:** * Medical University of South Carolina, Department of Emergency Medicine, Charleston, South Carolina; † Medical University of South Carolina, College of Medicine, Charleston, South Carolina

**Keywords:** case report, trigeminocardiac reflex, facial trauma, bradycardia

## Abstract

**Introduction:**

The trigeminocardiac reflex (TCR), a physiologic response to irritation of the branches of the trigeminal nerve, was first described in humans in 1870. Gastric hypermotility, hypotension, bradycardia, and even asystole have been reported in response to surgical manipulation of the trigeminal nerve and its branches, but literature is limited in patients not undergoing surgery. Although effects are generally transient and benign, TCR can present a significant diagnostic and therapeutic challenge in patients undergoing surgical manipulation of the trigeminal nerve and its branches.

**Case Report:**

We describe a case of severe bradycardia secondary to facial trauma causing hemodynamic compromise and diagnostic uncertainty.

**Conclusion:**

This case highlights a possible case of TCR, as well as therapeutic considerations, in a patient presenting to the emergency department with severe facial trauma.

## INTRODUCTION

The trigeminocardiac reflex (TCR) is defined as the sudden onset of parasympathetic dysrhythmia, hypotension, apnea, or gastric hypermotility during stimulation of the sensory fibers of the trigeminal nerve. It manifests during craniofacial surgeries or secondary to trauma and can present as an imbalance between sympathetic and parasympathetics nervous systems.^1^ This neurologic response to stimulation of the trigeminal nerve was first recognized in 1870, and the term “trigeminocardiac reflex” was coined in 1999 by Schaller et al.^2^ The exact mechanism, although unclear, is thought to be a protective one that aims at reducing cerebral blood flow and intracranial pressure. There are different clinical subtypes of TCR that present with various cardiorespiratory manifestations such as bradycardia, hypotension, apnea, and sometimes even the opposite sympathetic effects, depending on where along the reflex arc the nerves are stimulated.^1^–^3^ The oculocardiac reflex, a subtype of the TCR, has been described in the emergency medicine literature.^4^ The trigeminocardiac reflex has been documented in instances of surgical manipulation, but there is a paucity of data with regard to traumatic injuries not undergoing surgical intervention. We report a case of a 57-year-old male exhibiting severe bradycardia after presenting with significant facial trauma from a syncopal episode, prompting the need for vigilant monitoring and ultimately resulting in an intensive care unit (ICU) admission.

## CASE REPORT

**Image 1:**
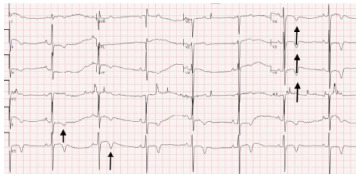
Electrocardiogram demonstrating sinus bradycardia and inferolateral T-wave inversions (arrows).

A 57-year-old male with past medical history significant for nonischemic cardiomyopathy, coronary artery disease, HIV, and hypertension presented to a Level I trauma center emergency department (ED) for a syncopal episode resulting in a ground-level fall while at a gas station. He experienced facial trauma from a syncopal event after prolonged bicycling on a hot day. On arrival to the ED, triage vital signs were notable for a heart rate of 49 beats per minute (bpm) and blood pressure of 142/95 millimeters of mercury (mm Hg). He was noted to be mildly confused and had significant facial trauma with a left-sided mandible deformity, intra-oral bleeding, chin and lip lacerations, and instability to his upper and lower teeth. Analgesia, tetanus prophylaxis, and fluid resuscitation were initiated. An electrocardiogram (ECG) (Image 1) demonstrated sinus bradycardia with a rate of 45 bpm and inferolateral T-wave inversions. Labs were notable for a lactate of 4.7 millimoles per liter (mmol/L) (reference range: 0.7–2.1 mmol/L) and creatinine 2.3 milligrams per deciliter (mg/dL) (0.7–1.3 mg/dL) with a baseline creatinine of 1.1 mg/dL. Electrolytes and troponin were within normal lab ranges.

**Image 2:**
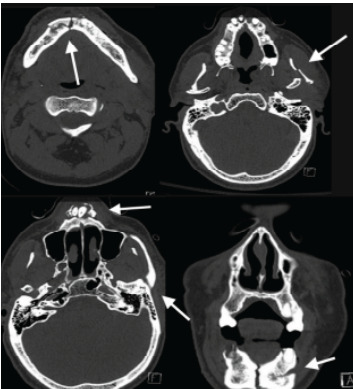
Maxillofacial computed tomography images of the patient’s multiple facial fractures, including a comminuted symphysis fracture, subcondylar fracture, and teeth intrusions (arrows)

Approximately two hours into the patient’s ED stay, while awaiting computed tomography (CT), he was noted to have episodes of symptomatic bradycardia to as low as 35 bpm with associated respiratory distress and nausea. These episodes were particularly pronounced with supine positioning, improving with upright positioning and suctioning of secretions. Given a high clinical concern for intracranial and/or maxillofacial injuries, it was decided to attempt CT while the ED staff closely monitored him. While in the CT scanner, he had two more episodes of bradycardia with heart rates of 20–30 bpm upon lying flat that resolved with upright positioning and suctioning. Given concerns for impending hemodynamic collapse and lack of airway protection, the patient was taken to a resuscitation bay and evaluated with the trauma service. A vagal-mediated bradycardia was suspected, and the patient was administered 1 mg of intravenous atropine with resolution of bradycardia. Non-contrast CT of the head revealed no intracranial injuries, and non-contrast CT of the facial bones (Image 2) demonstrated bilateral subcondylar fractures, comminuted symphysis fracture, fracture of anterior maxilla with intrusion of teeth #7 – 10, and multiple skin lacerations.

*CPC-EM Capsule*

What do we already know about this clinical entity?

*The trigeminocardiac reflex (TCR) is a physiologic response to irritation of branches of the trigeminal nerve and has been described in craniofacial surgery literature*.

What makes this presentation of disease reportable?

*To date, TCR has not been described in response to maxillofacial trauma independent of surgical manipulation*.

What is the major learning point?

*Clinicians should keep TCR in their differential diagnosis when faced with characteristic hemodynamic instability in patients with facial trauma*.

How might this improve emergency medicine practice?

*Awareness of TCR will aid emergency physicians in recognizing and managing this potentially life-threatening neurologic response*.

The patient was admitted to the trauma ICU for airway and cardiac monitoring with plans for open reduction and internal fixation of his injuries. During his admission, he continued to suffer from episodes of severe sinus bradycardia. The patient was evaluated by cardiology for possible pacemaker placement in the setting of bradycardia. Per chart review, the patient’s baseline heart rate was 60–75 bpm (no prior ECGs are available), and he was not prescribed atrioventricular (AV) nodal blocking agents. Ultimately it was determined that the patient’s bradycardia was mediated by increased vagal tone secondary to facial trauma. On hospital day 2, he underwent surgical fixation of his injuries with no reported significant intraoperative events. With surgical fixation on hospital day 2 the patient had no further episodes of bradycardia and was uneventfully discharged on hospital day 5.

## DISCUSSION

The trigeminocardiac reflex is a rare phenomenon that has been described during maxillofacial surgery. Stimulation of the trigeminal nerve sends an afferent signal to the brainstem, which manifests in efferent vagal nerve-mediated modulation of blood pressure, inducing bradycardia. Much of the current literature on the topic describes its occurrence and management in maxillofacial surgery. Although the oculocardiac reflex has been described in relation to ocular trauma, this case provides a unique addition to the literature, as our patient did not sustain ocular trauma. If our case was indeed a TCR-mediated bradycardia, it would provide valuable insight into expansion of the current definition of TCR. The incidence of TCR during maxillofacial and cranial surgery is estimated to be 11–18%.^5^,^6^ Clinicians must be aware of this phenomenon as it can occur in the absence of known cardiac conduction disease (eg, sinus node dysfunction, AV block), and close monitoring should be provided.

Suspicion of TCR in this patient was largely based on elimination of other causes for his bradycardia and symptoms. Proposed diagnostic criteria for TCR include the following: characteristic changes in heart rate (10–20% drop) and blood pressure; plausibility of a relationship to irritation of trigeminal nerve; and repeatability.^2^ The patient was not taking any AV nodal blocking and did not have a known history of bradycardia. He sustained no other significant trauma beside his mandibular and maxillary fractures that did not involve the orbit. The bradycardic episodes were also repeatable with supine positioning. The bradycardic episodes were >20% decrease from his baseline. Of note, the patient had no significant recorded hypotension. Our suspicions were further supported by the improvement of his bradycardia with atropine and its resolution with surgery. While improvement with atropine is not unique to TCR, it does suggest a vagally mediated bradycardia. The lack of bradycardic episodes following surgery thus gave us high suspicion that the TCR was the cause of his bradycardia. Finally, the patient was evaluated by the electrophysiology service, which concluded he most likely suffered bradycardic episodes related to heightened vagal tone consistent with the TCR.

While published data on the treatment of TCR specifically is sparse, much can be applied from the treatment of other bradycardias, specifically vagal-mediated bradycardias. The surgery literature suggests consideration of prophylactic treatment with atropine or epinephrine.^6^ The use of hemodynamic-altering medication, however, may cloud the clinical picture in the ED. Initial management includes ensuring airway patency, oxygenation, and establishing IV access. Noxious stimuli and repositioning can improve the heart rate, as was seen in our patient. External cardiac-pacing pads should be at the bedside, ready to be applied. The primary pharmacological treatment of TCR is atropine, administered as 1 mg intravenously every 3–5 minutes, up to a total dose of 3 mg. Epinephrine and transcutaneous or transvenous pacing can also be considered as additional therapy in cases refractory to other treatments.^7^ Finally, we recommend expert consultation with the trauma and cardiology services in severe or refractory cases.

While this case report provides valuable insights into the potential for TCR to occur in instances beyond surgical manipulation, it is important to acknowledge its limitations. Most of the supporting literature discusses this reflex primarily in the context of surgical manipulation, with limited reference to facial trauma. Variability in injury severity, patient physiology, and underlying electrophysiologic conditions may limit the generalizability of our conclusions. Despite these limitations, we suggest that clinicians consider TCR in the context of facial trauma, where mechanical stimulation of the trigeminal nerve can lead to potentially life-threatening bradycardia, hypotension, or cardiac arrest.

## CONCLUSION

The trigeminocardiac reflex is a brainstem-mediated response triggered by stimulation of the trigeminal nerve or its branches, leading to parasympathetic activation. This reflex arc can result in bradycardia, hypotension, and even asystole. It is particularly common during manipulation of orbital or maxillofacial structures in surgery, but as reported here, it may be seen in maxillofacial trauma. Management of TCR-induced bradycardia involves a systematic approach to stabilize the patient and address the underlying cause. We suggest that clinicians become aware of TCR and its treatment and to remain vigilant that it may present outside the surgical theater.
